# The Immunomodulatory and Anti-Inflammatory Effect of Curcumin on Immune Cell Populations, Cytokines, and In Vivo Models of Rheumatoid Arthritis

**DOI:** 10.3390/ph14040309

**Published:** 2021-04-01

**Authors:** Sebastian Makuch, Kamil Więcek, Marta Woźniak

**Affiliations:** 1 Department of Pathology, Wroclaw Medical University, 50-367 Wroclaw, Poland; sebastian.mk21@gmail.com; 2 Department of Biotechnology, Wroclaw University, 50-383 Wroclaw, Poland; kamil.wiecek24@gmail.com

**Keywords:** rheumatoid arthritis, curcumin, immune cells, immunomodulation, autoimmune disease

## Abstract

Rheumatoid arthritis (RA) is a widespread chronic autoimmune disorder affecting the joints, causing irreversible cartilage, synovium, and bone degradation. During the course of the disease, many immune and joint cells are activated, causing inflammation. Immune cells including macrophages, lymphocytes, neutrophils, mast cells, natural killer cells, innate lymphoid cells, as well as synovial tissue cells, like fibroblast-like synoviocytes, chondrocytes, and osteoclasts secrete different proinflammatory factors, including many cytokines, angiogenesis-stimulating molecules and others. Recent studies reveal that curcumin, a natural dietary anti-inflammatory compound, can modulate the response of the cells engaging in RA course. This review comprises detailed data about the pathogenesis and inflammation process in rheumatoid arthritis and demonstrates scientific investigations about the molecular interactions between curcumin and immune cells responsible for rheumatoid arthritis development to discuss this herbal drug’s immunoregulatory role in RA treatment.

## 1. Introduction

Rheumatoid arthritis (RA) is one of the most widespread chronic inflammatory diseases, affecting about 1% of the total world population [1,2,3], with yet unknown etiology. This autoimmune disorder is characterized by burdensome pain, swelling, and, usually, stiffness of symmetrical joints of hands, wrists, feet, and knees, greatly reducing mobility and overall comfort of life. During RA progression, persistent inflammation forces systematic changes causing irreversible cartilage, synovium, and bone degradation, finally deforming the whole joint structure, leading to loss of mobility and muscle atrophy [1,4,5]. This disease mostly affects joints; however, in approximately 40% of patients, it can also cause extraarticular manifestations of many sorts forming rheumatic nodules, causing lungs and blood vessel diseases or lead to anemia, peripheral neuropathy, and disorders in many different organs [4,6] (what is interesting, despite the fact that women are two to three times more likely to develop RA, they are less prone to develop extraarticular symptoms [2]). Moreover, rheumatoid arthritis features include loss of weight, fatigue, and fever, which, together with other manifestations, may lead to disability or even premature death [1,5,7].

During the course of the disease, a complicated network of relations is being established, self-perpetuating more and more aggressive inflammation. In sick patients, the joint maintains an unending conflict between anti- and proinflammatory factors with the second group’s dominance. After earlier initiation of an autoimmunologic reaction and inflammation establishment, leucocytes gradually infiltrate a joint due to the attractive properties of circulating chemokines, loosening of cartilage structures, and ongoing angiogenesis [8]. At the same time, fibroblast-like synoviocytes (FLS), which form a part of the synovium, change their functioning, hyperproliferating and releasing other disease-intensifying factors, including those involved in osteoclastogenesis. Due to the activity of activated chondrocytes and metalloproteinases released by synoviocytes, the joint cartilage is damaged. Degradation of mineral and non-mineral bone elements occurs mostly due to osteoclast- and synoviocyte-released cathepsin K activity [6,9,10].

A significant number of studies have reported the tremendous potential of medicinal plants and compounds isolated from them in the comparative and alternative treatment of autoimmune diseases. The combination of standard protocols or novel therapies with natural plants’ extracts is an auspicious approach. This review summarizes the up-to-date and detailed information about the pathogenesis and inflammation process occurring in rheumatoid arthritis and describes the most substantial cells and cytokines involved in the pathological course of RA. Furthermore, it sheds light on the potential immunomodulatory role of curcumin (diferuloylmethane), a natural compound from the root of turmeric (*Curcuma longa*), in this autoimmune disorder. Due to an incredible amount of studies about curcumin, this phytochemical is known to have an impressive number of therapeutic properties. Herein, we collected scientific investigations on various in vitro and in vivo models of inflammation, demonstrating the effect of curcumin on cells and the cytokines secreted by them and involved in the pathogenesis of rheumatoid arthritis.

## 2. Understanding the Pathogenesis of RA and the Effects of Curcumin

To better understand how RA affects human bodies, we need to know the synovium’s composition and function. The synovial membrane (synovium) is built by two layers composed of the intima and the underlying subintima layer. Subintima is mainly formed by vascularized connective tissue containing collagen fibers and evenly dispersed fibroblast- and macrophage-like synoviocytes (FLS and MLS). In a healthy synovium, this area normally has very few cells. Intima is a thin layer (1–2 cells-thick) composed mainly of FLS intercalated with MLS. The primary function of the synovium is to maintain joint homeostasis by regulating the synovial cavity influx and efflux. In this system, the intimal lining plays a crucial role—its loose fit allows the flow of essential substances and cells. Not only is the synovium responsible for regulating transport through the membrane, but its products are also vital for entire joint functioning. Fibroblast-like synoviocytes synthesize joint lubricants such as hyaluronic acid, oversee synovial fluid volume, regulate immunological processes, maintain extracellular matrix, secret hyaluronan, and clean intraarticular debris [11].

There are no direct causes that could lead to rheumatoid arthritis development because the disease itself seems to be of heterogeneous origin. RA appears to be more like a set of different (patient-dependent) but linked diseases leading to common outcomes. Possible proof for that statement may be found in the presence or absence of RA markers (i.e., specific antibodies) and differences in their levels, varying between patients at different stages of the disease [12]. The predisposition to RA development is about 60% hereditary. From 11% up to 37% of cases, it depends on the functioning of specific alleles of the human leukocyte antigen (HLA), which is involved in forming a major histocompatibility complex class 2 (MHCII) [1,6,13].

Besides possible genetic predispositions, a major role in RA development may be played by environmental factors such as diet, smoking, or contact with certain microbes (like *Porphyromonas gingivalis*), which can impact the patient’s immune system, mainly by increasing the number of autoantibodies.

Another important aspect is the role of epigenetic modifications in rheumatoid arthritis. DNA methylation, abnormal expression of non-coding RNAs, and cell type-specific histone modifications have been linked with RA [14,15]. It has been reported that hyperacetylation of histone H3 in the IL-6 promoter triggers the increase of IL-6 production by rheumatoid arthritis synovial fibroblasts (RASFs) [16]. Another study has shown that the extent of total histone H3 acetylation in peripheral blood mononuclear cells obtained from RA patients was increased compared to healthy controls [17]. Moreover, the balance between histone acetyltransferase (HAT) and histone deacetylase (HDAC) activity has been found to be significantly shifted towards histone acetylation in RA synovial tissue [18]. In 2015, a genome-wide study revealed the significant role of DNA methylation in lymphocyte populations obtained from RA patients [15,19]. Thus, the association between epigenetic modifications and pathogenesis of RA is indisputable.

Interestingly, many studies have described the potential role of curcumin as an epigenetic modifier. This potent herbal drug has been identified as an inhibitor of DNA methyltransferases (DNMTs), regulator of histone acetyltransferases (HATs), deacetylases (HDACs), and microRNAs, as well as a DNA binding agent [20]. Curcumin has been found to significantly reduce H3ac levels in the IL-6 promoter as well as IL-6 mRNA expression in rheumatoid arthritis synovial fibroblasts (RASFs) [16]. Even though the role of curcumin as an epigenetic modifier has been well documented in cancer, neurological disorders, and some inflammatory diseases [21], only a limited amount of in vitro and in vivo studies have been performed to establish the precise epigenetic regulatory effects of this herbal drug on RA models.

**Figure 1:**
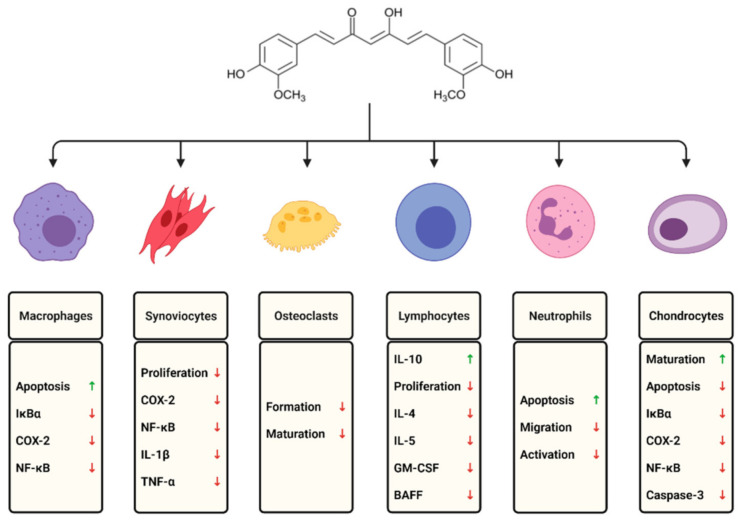
The effects of curcumin on selected immune cells involved in the course of rheumatoid arthritis.

Although there is no cure available for rheumatoid arthritis, currently, the research is aimed at minimizing inflammation, pain, and joint damage, enhancing remission of symptoms, and improving the quality of life. Many studies have found that curcumin has a prominent effect on different immune cells and inflammatory mediators (Figure 1).

Curcumin is a polyphenolic substance naturally occurring in turmeric, especially in *Curcuma Longa*, with broad anti-inflammatory properties and proven positive effects in autoimmunological disease therapies, including RA. Curcumin is an antioxidant, which means it can efficiently reduce the level of reactive oxygen species (ROS), weaken redox signaling, and reduce inflammation [22]. In addition to having direct antioxidant properties, curcumin also blocks the activity of ROS-generating enzymes like lipoxygenase (LOX), cyclooxygenase (COX), xanthine dehydrogenase, and nitric oxide synthase (iNOS) [13,23,24]. Despite reducing ROS levels, curcumin also possesses numerous other properties that enable its usage as a potential therapeutic drug targeted against RA. Interesting insights into this matter are provided by recent studies, which found that this natural compound can suppress proinflammatory pathways related to the immune cells crucial in RA development. Therefore, curcumin’s daily consumption can decrease inflammation and oxidative stress, contributing to the immune system’s modulation and alleviating the rheumatoid arthritis course.

Besides beneficial properties, curcumin per se exhibits very low ADME (absorption, distribution, metabolism, excretion) scores, making it a challenging compound to use in potential therapies [25,26]. Several studies have shown that this polyphenol has a poor pharmacokinetic profile [27,28]. Under physiological conditions, curcumin is prone to undergo degradation via solvolysis and autoxidation, reduction by various enzymes and conjugation (glucuronidation or sulfonation), leading to loss of native values and being eliminated from the system [25,26,29]. Furthermore, curcumin’s low bioavailability and solubility in aqueous media, as well as susceptibility to photodegradation, makes it even more problematic [25]. To mitigate the abovementioned disadvantages, various vehicles such as formulations based on liposomes, phospholipid complexes, emulsions, or colloidal nanoparticles can be used [26]. A clinical trial evaluating an innovative and highly bioavailable formulation of curcumin showed significant analgesic and anti-inflammatory properties which relieved the symptoms of rheumatoid arthritis [30]. Nevertheless, novel formulations of curcumin and additional clinical trials on RA patients are still in dire need.

In the next section, the link of rheumatoid arthritis immune cells with curcumin’s anti-inflammatory properties is discussed. Subsequently, the specific impact of this plant derivative on the different immune cell responses is elaborated.

## 3. Cells Involved in the Course of RA and the Modulatory Role of Curcumin

### 3.1. Macrophages and Monocytes

Most RA macrophages are marrow- and monocyte-derived and exhibit an aggressive proinflammatory phenotype, changed due to exposure to many factors like FLS-released cytokines and prostaglandins, T cell interactions, immune complexes, etc. [31,32]. Highly activated macrophages are among the key factors in progressing RA, mostly due to their expression profile. They are responsible for releasing heparin-binding EGF-like factor, TNF-α, chemokines (MCP-1/CCL2), vasoactive peptides, digestive enzymes (collagenases, MMPs), prostaglandins, interleukins (IL-1, IL-6, IL-12, IL-15, IL-18, IL-23), reactive oxygen and nitrogen intermediates. RA macrophages also contribute to phagocytosis and antigen presentation, stimulating antigen-specific T cell proliferation and production of pro-inflammatory mediators in B cells [1,11,31,32,33].

To investigate whether curcumin can be therapeutically beneficial for macrophages in inflamed joints, Wang et al. used LPS-activated RAW264.7 macrophages as model cells to test the hypothesis. Flow cytometry and terminal deoxynucleotidyl transferase dUTP nick end labeling (TUNEL) assay results proved that curcumin in vitro enhanced macrophage apoptosis. Moreover, in curcumin-treated cells, the IκBα level in the cytoplasm was decreased; this correlates with reduced expression of COX-2, further confirming that curcumin exerts anti-inflammatory activity by inhibiting NF-κB activation [34]. This statement is also consistent with previous research explored by Murakami et al. [35].

### 3.2. Lymphocytes

Lymphocytes form a major yet heterogeneous group of cells infiltrating RA synovium. Therefore, it is quite challenging to determine the exact function of each subpopulation. We may differentiate several subgroups of CD8+ effector, TH1, TH2, Th17, Treg, and Tph lymphocytes from T cells. TH1 lymphocytes express proinflammatory cytokines, mostly TNF-α and IFN-γ, whereas TH2 lymphocytes produce anti-inflammatory mediators. However, a disbalance in the activity of those populations leads to the enhancement of the disease. Recently discovered Tph lymphocytes, which share common elements with T follicular helper cells (Tfh), have shown in vitro ability to induce B cell differentiation into plasma cells and expression of IL-21 and CXCL12. Th17 cells seem to play a vital role in the development of RA due to a broad spectrum of secreted cytokines and their impact on different cells. This population of T helpers is capable of producing IL-17A, IL-17F, IL-21, IL-22, and TNF-α, GM-CSF (granulocyte-macrophage colony-stimulating factor), and IFN-γ [12,31,32]. Furthermore, they may be involved in activation of FLS, maturation and function of osteoclasts (i.e., via upregulating RANKL expression), recruitment and activation of B cells, macrophages, and neutrophils in RA. Moreover, they may probably alter the glycosylation profile of antibodies secreted by plasmablasts even before the beginning of the clinical phase of the disease [1,12,31,32].

Synovial tissues affected by RA contain B cells mostly in aggregates forming clusters resembling tertiary lymphoid follicular structures with germinal centers, promoting immunoglobulin class switching thus locally generating plasma cells from activated B cells [32]. RA milieu supports B cell functioning by a rich pool of cytokines necessary for their survival like a proliferation-inducing ligand (APRIL), B lymphocyte stimulator (BLyS), or IL-6 [31,33].

This natural compound can modify lymphocyte activation through downregulation of proinflammatory cytokines in many pathological conditions whose pathophysiology is related to inflammation. Research indicates that curcumin inhibits lymphocyte proliferation and the cells’ ability to secrete IL-4, IL-5, and the granulocyte-macrophage colony-stimulating factor (GM-CSF). Furthermore, curcumin can enhance production of anti-inflammatory IL-10 to counteract inflammatory conditions [36]. Another role of curcumin in the modulation of B lymphocytes was investigated by Huang et al. The in vitro experiments on collagen-induced arthritis (CIA) in mice showed that curcumin significantly inhibited IFN-γ-induced BAFF expression through suppression of signal transducers and activators of transcription 1 (STAT1) signaling. The studies stated that decreased BAFF expression may be a novel mechanism by which this natural derivative improves RA [37].

### 3.3. Neutrophils

Despite acting in different types of inflammation by secreting proinflammatory cytokines, chemokines, etc., neutrophils also contribute to disease development and persistence uniquely by releasing cytotoxic products and promote nucleotide signaling through the release of ATP by neutrophil-secreting vesicles [38]. Another manner in which neutrophils can stimulate RA is their ability to generate neutrophil extracellular traps (NETs). These structures expose autoantigens, which in turn increases autoantibody production. Synovial B cells are highly reactive to citrullinated proteins, including the histones exposed in the released chromatin, which leads to additional autoantibody production. What is more, anti-citrullinated protein antibodies can stimulate NETosis creating a vicious circle of self-perpetuating proinflammatory response in RA [1,12,33].

The detailed impact of curcumin on neutrophils in a mouse model of arthritis was studied by da Silva et al. The researchers investigated the effects of a nanoencapsulated association of curcumin and vitamin D3 on purine metabolism enzymes in neutrophils. They revealed it decreased infiltration and activation of neutrophils stimulated by curcumin in animals [39]. Jacksons et al. also suggest that curcumin inhibits the migration of neutrophils from human blood to inflamed joints and acts as a proapoptotic agent in the treatment of crystal-induced arthritis or rheumatoid arthritis [40].

### 3.4. Natural Killer (NK) Cells

NK population can be distinguished into two subpopulations of cytotoxic CD56dim and CD56bright cells producing proinflammatory cytokines such as TNF or IFN-γ. During the early stages of RA, the population of CD56dim cells is decreased, which possibly contributes to enhanced survivability of different proinflammatory cell populations such as activated T cells. Additionally, CD56bright subpopulation in both RA synovium and synovial fluid is increased and presents a higher activity than peripheral blood cells. Cytokines expressed by NK cells may support dendritic cells’ maturation and further disease progression by TNF-mediated osteoclastogenesis and possibly via IFN-γ-dependent naïve T cell polarization into Th1 and Treg suppression [32,41,42,43]. However, according to the newest publications, the CD56bright subpopulation may actually not be composed of NK cells but of ILC1s (innate lymphoid cells) to which (depending on classification) NK belong [44].

Little is known about the modulatory effect of curcumin on NK cells, cytotoxic T cells, or other innate lymphoid cells in autoimmune disorders [45]. However, many studies show that curcumin has an immunostimulatory effect on NK cells by increasing the surface expression of the CD16+ and CD56dim population of NK cells in aggressive cancers, especially in breast cancers [46,47]. The number of NK cells and their cytotoxic effect on cancer cells or in an experimental autoimmune myasthenia gravis model [48] is significantly enhanced in the presence of curcumin.

### 3.5. Fibroblast-Like Synoviocytes

Fibroblast-like synoviocytes also known as type B synoviocytes play a crucial role in RA. They do not form a homogeneous population and may vary even between joints. After activation, they change their phenotype similarly to tumor cells, losing contact inhibition and anchorage dependence, proliferating, gaining migratory activity and expressing large quantities of cytokines, chemokines, RANKL (receptor activator of nuclear factor kappa-β ligand), adhesion molecules, MMPs, and TIMPs (tissue inhibitor of metalloproteinase). FLS promote the influx of leukocytes, which results in chronic synovitis after their stimulation. FLS play a significant role in the destruction of both cartilage and bone tissue. Cartilage destruction is driven by FLS mainly by releasing MMPs, activation of chondrocytes and increasing tissue catabolism. Bone loss proceeds due to FLS-forced increase of osteoclastogenesis stimulated by upregulated M-CSF (macrophage colony-stimulating factor), increased RANKL expression, and increased cathepsin K activity [9,11,33,49].

The most common cell type in rheumatoid arthritis is fibroblast-like synoviocytes. The joint destruction by their massive production of chemokines, cytokines, and matrix-degrading enzymes can be effectively reduced by curcumin. Many studies confirm that this polyphenol can induce apoptosis by activating proapoptotic proteins and downregulation of Bcl-2, Bcl-xL, and poly(adenosine diphosphate (ADP)-ribose) polymerase in the synovial fibroblasts from patients with RA [50,51]. It may also reduce the oxidative damage in the synovial fluid and the inflammatory response by improving the production of IL-10 [52].

The experiment conducted by Moon et al. presented curcumin’s anti-inflammatory properties in synovial fibroblasts, manifested by suppression of COX-2 and subsequently blockade of prostaglandin E2 synthesis [53]. Dai et al. also confirmed that this natural compound reduces inflammation, synovial cells hyperplasia, and other main features of CIA-induced RA in rats via the mTOR pathway [54]. Moreover, curcumin acts by inhibiting synovial cell proliferation and downregulation of various nuclear factor kappa B complexes, IL-1β, and TNF-α [55].

### 3.6. Mast Cells

Nowadays, mast cells (MC) are considered to be involved in RA development. MCs have a great variety of releasing mediators. In vitro and animal model-based research showed that they can produce both immunomodulating cytokines such as IL-10, which stomps immune response, and those that activate B cells to reinforce inflammation. According to some recent research, mast cells may be associated with RA, especially during the early development stage. IgG immune complexes may activate MCs to produce various proinflammatory cytokines, which collectively can inhibit FLS apoptosis, promote angiogenesis, enhance B cell survival, proliferation, and increased IgG secretion. For instance, by way of CD40L–CD40 interaction, they activate FLS collagenases and participate in neutrophil recruitment (directly and indirectly via tryptase release) and increase osteoclast activity. Additionally, MCs can probably mobilize T cells by presenting antigens (MHCII) and interact with tertiary lymphoid organs [56,57,58,59,60]. Still, there are many unknowns concerning RA mast cells’ function because their behavior and expression profiles may significantly vary between rheumatoid arthritis phenotypes and stages of the disease itself. Therefore, further insightful research is necessary for a better understanding of their potential pathogenic role.

Regulation of cytokine secretion from mast cells by curcumin is also an important therapeutic strategy for inflammatory diseases. Kong at al. suggest that bisdemethoxycurcumin (BDCM), one of the significant components of *Curcuma longa* L., can inhibit the expression of inflammatory mediators by suppressing NF-κB activation in human mast cell line 1, HMC-1. This substance inhibits the ERK, JNK, p38 MAPK, and NF-κB pathways [61]. Furthermore, this natural polyphenol inhibits connective tissue type mast cells (canine cutaneous mastocytoma mast cells (CM-MC)) by activation of degranulation, calcium mobilization, intracellular ROS generation, cell membrane lipid peroxidation, and tyrosine/threonine phosphorylation [62]. These results implicate curcumin as a valuable natural compound that modulates mast cell-mediated inflammation in RA.

### 3.7. Chondrocytes

Cartilage is a tissue composed of specific cellular matrix and resident chondrocytes. Under normal circumstances, chondrocytes are responsible for maintaining proper cartilage composition by destroying redundant molecules and secreting new. In an RA cell- and cytokine-rich milieu, chondrocytes are activated (for example, via CXCL6 or FGF2 stimulation or in NF-kB- or STAT3-dependent manner [63]) and change their expression profile by decreasing synthesis of matrix-forming proteins and producing tissue-destroying enzymes, especially MMPs and ADAMTS (a disintegrin and metalloproteinase with thrombospondin motifs) [64]. Furthermore, some RA mediators like IL-1 and IL-17 may cause chondrocyte apoptosis [65], which, together with enzyme-driven matrix degradation, causes severe cartilage destruction.

In chondrocytes, curcumin acts by inhibiting the proinflammatory process, suppressing apoptosis, and supporting chondrogenesis. Even a very low concentration of curcumin (0.5 μM) inhibits IL-1β-induced IκBα phosphorylation and NF-κB nuclear translocation, activation of caspase-3 and cyclooxygenase-2 in mesenchymal stem cells (MSCs, isolated from canine adipose tissue biopsies) and in primary canine chondrocytes isolated from cartilage. This action provides a suitable microenvironment for MSC-like progenitor cells to undergo chondrogenesis, which may support cartilage regeneration in RA [66]. Furthermore, when curcumin was added to cultures of human articular chondrocytes treated with IL-1 beta and TNF-alpha for up to 72 h, this natural compound inhibited IL-1β and TNF-α-mediated collagen type II and beta 1 integrin degradation and thus led to the recovery of cartilage-specific matrix components in chondrocytes [67].

### 3.8. Osteoclasts

Progressing bone degradation in RA is caused mainly by a disbalance between an impaired function of osteoblasts and hyperactive osteoclasts. Activated osteoclasts attach to the bone surface and, via proton pumps, acidify the local environment. As a result, bone demineralization and secretion of enzymes such as cathepsin K and MPPs are observed. Inflamed synovial tissue contains many molecules that enhance osteoclastogenesis (primarily RANKL, TNFα, IL-6, IL-17), promote osteoclast activity and survival (IL-1, prostaglandin E2), or inhibit osteoblasts’ differentiation via Wnt inhibitors like DKK1. Interestingly, anti-citrullinated protein antibodies and IgG immune complexes can stimulate different cells to produce proinflammatory and proosteoclastogenic cytokines like TNF-α and directly interact with osteoclast progenitors via fragment crystallizable gamma (Fcγ) receptors promoting their further differentiation [68,69].

Some studies demonstrate that curcumin has a significant impact on osteoclasts. Shang et al. revealed that curcumin inhibits osteoclast formation via preventing the phosphorylation of components of the MAPK signaling pathways. The authors used peripheral blood mononuclear cells (PBMCs) from patients with rheumatoid arthritis and incubated cells with different curcumin concentrations (2.5, 5, 10 μM) for 48 h. The results show that curcumin inhibited the number of osteoclasts generated from PBMCs in a dose-dependent manner. Moreover, curcumin inhibited M-CSF- and RANKL-stimulated osteoclast differentiation via the suppression of ERK1/2, p38, and JNK activation and the inhibition of c-Fos and NFATc1 expression [70]. Other authors evaluated the capacity of curcumin (1 and 10 μM) for human osteoclastogenesis inhibition (on the osteoclasts generated from peripheral blood mononuclear cells) and showed that this natural plant derivative abrogated both osteoclast differentiation (by 56 and 81%) and bone-resorbing activities (by 56 and 99%). These results were accompanied by the inhibition of IκB phosphorylation, which led to NF-κB deactivation [71]. Both studies concluded that curcumin may be a potential therapeutic agent for bone deterioration treatment in RA.

## 4. RA Markers and Most Common Cytokines—Potential Therapeutic Targets

For many years, before chronic and severe development, an increase in specific antibodies in a patient’s peripheral blood can be detected [5,12]. The aforementioned anti-citrullinated protein antibodies and rheumatoid factor belong to the most common RA-associated markers. Due to its autoimmune properties, RA development is controlled mainly by the local distribution of different proinflammatory factors, including many cytokines, angiogenesis-stimulating molecules, etc. In this section, the most common cytokines and their role in rheumatoid arthritis are described.

In particular, IL-17 produced by T helper cells 17 (Th17) seems to be crucial because it has been shown that this cytokine is involved in the regulation of expression of many different cytokines and chemokines involved in developing inflammation, metalloproteinases (MMP) degrading cartilage tissue, prostaglandin E2, and cyclooxygenase 2. Moreover, IL-17 promotes ligand RANK (RANKL) expression at the surface of cells, which causes transformation of monocytes to bone-degrading osteoclasts [1,72].

### 4.1. IL-1 Family

The IL-1 cytokine family consists of 11 subtypes that participate in innate and adaptive immune responses and vary in pro-and anti-inflammatory activities [73]. In RA, IL-1α, IL-1β, IL-18, and IL-33 are highly expressed and seem to fulfil a role in exacerbating disease progression [74,75]. In a narrower scope, IL-1 cytokines mainly apply to IL-1α and IL-1β subtypes, whereas IL-18 and IL-33 are distinguished separately.

### 4.2. IL-1

IL-1 cytokines can participate in the transduction of many molecular pathways (for instance, in the NF-kB- or AP-1-related manner [76]), inducing different molecules’ production. Those capabilities can be extremely significant in RA because IL-1 activity leads to stimulation of cells (especially of synovial fibroblasts), increasing production of cytokines, chemokines, and inducible nitric oxide synthase (iNOS), prostaglandins, MMPs, GM-CSF [74,76,77], and adhesion molecules of endothelial cells [78]. Those cytokines are also involved in osteoclast activation [62], for example, through Wnt signaling blockade. IL-1 presents many synergistic activities; together with TNF-α, IL-1 induces dickkopf-related protein 1 (DKK1) and sclerostin (SOST), which are the Wnt/β-catenin signaling antagonists. Due to the Wnt canonical pathway blockade, osteoblast differentiation decreases cooperatively with the downregulation of osteoprotegerin (OPG) and increased RANKL expression [79]. Additionally, IL-1β with IL-6 promotes Th17 differentiation. Anti-IL-1 therapies are already in use and help in the reduction of cartilage and bone damage. However, effects obtained in human patients present less efficiency than in animal models [76]. Experiments conducted on animal models of RA show that curcumin alleviates production of IL-1β, making it a promising potential drug [54,55].

### 4.3. IL-18

Predominantly IL-18 was mostly associated with enhancing IFN-γ production [80] in IL-12- or IL-15-related mechanisms [81]; nevertheless, this cytokine may participate in many other processes. Moreover, IL-18 is involved in inhibition of chondrocyte proliferation and activation of T cells (for instance, Th1, γδ T), NK cells, and macrophages [78,82]. Additionally, IL-18 induces the production of IL-17 [82], IL-32 [75], IL-2, IL-2Rα, TNF-α, GM-CSF, prostaglandin E2, MMP3, chemokines [78], adhesion molecules, and angiogenic factors [82]. In murine models of RA, mice with knockdown of IL-18Rα (IL-18 receptor α) presented decreased levels of IL-6, IL-18, TNF-α, IFN-γ, and MMP3, as well as less severe disease progression [83], which can implicate the importance of this cytokine in the course of this affliction. A research study published by Yin et al. revealed that curcumin suppressed the secretion of IL-18 in mouse bone marrow-derived macrophages [84]. Other, trials performed in murine and cell models of different diseases (including osteoarthritis) implicate that curcumin decreases expression of IL-18, but this topic has not been explored with the usage of RA models [85,86].

### 4.4. IL-33

The last cytokine belonging to the IL-1 family is IL-33. IL-33 stands in a peculiar position in RA due to uncertainty about whether it contributes to disease development or helps relieve it. On the one hand, IL-33 works as a neutrophil attractor, induces mast cell activation, and promotes secretion of various molecules such as chemokines and cytokines (e.g., IFN-γ, TNF-α, IL-6, IL-1β), and probably indirectly enhances immunoglobulin production by B cells and mast cell degranulation via an IL-5- and IL-13-dependent way by stimulating CD4+ T cells [87,88,89]. On the contrary, in some models, it has been shown that IL-33 silencing suppresses TNF-α-induced production of prostaglandin E2 and reduces IL-6, IL-8, and MCP-1 levels [78]. That ST2 (IL-33 receptor) blockade attenuates RA severity and decreases the production of IL-17, IFN-γ, and RANKL mRNA [88]. Furthermore, IL-33 promotes Th2 response boosting up IL-4, IL-5, and IL-13 [87] production, triggers type 2 innate lymphoid cells (ILC2) and Treg expansion, and leads to macrophages’ polarization into the regulatory M2 phenotype [90] and decreases their osteoclast differentiation [89]. Additionally, IL-33-ST2 binding can downregulate the Toll-like receptor (TLR)-induced immune response by competing for the use of the MyD88 protein [88]. This possibly attenuating IL-1-related molecular answers to similarities between the TIR signaling pathway (Toll/interleukin-1 receptor) and the TLR signaling pathway. The acquired results may give opposite effects due to differences in the used models, stages of the disease, and whether IL-33 itself or its receptor was tested [89]. It has been shown that curcumin may suppress the expression of IL-33; however, it was proved neither in RA patients nor in any models [91].

### 4.5. IL-6 and IL-27

IL-6, together with IL-27, belongs to a broader group of cytokines, which can act in a pro-and anti-inflammatory manner [92,93] mediating lipid and iron metabolism, regulating overall levels of organisms’ fatigue and pain, stimulating angiogenesis and disease progression [94,95]. In RA development and course, IL-6 seems to be another pivotal cytokine and is already targeted in various therapies [96]. During the course of the disease, IL-6 plays a major role in controlling local cellular composition, functioning, and survivability. IL-6, together with TNF-α, stimulates growth and activation of FLS, which express different inflammation-sustaining cytokines and further participate in the increased production of IL-6 [96]. In addition, IL-6 can enhance pannus formation by increasing VEGF expression [49]. Furthermore, IL-6 is involved in increasing oxidative stress [49], promoting mononuclear cell infiltration [92], and activation of lymphocytes [96]. Activated B cells differentiate into plasma cells and increase antibody levels, whose production and class switching may also be affected by this cytokine [93]. Additionally, IL-6-driven B cell differentiation can impact bone changes via increasing DKK1 expression and blocking Wnt signaling [79]. IL-6 is also a critical factor in overseeing T cell-mediated acute immune responses by advancing their differentiation with emphasis on Th17 cell expansion [93] and inhibition of Treg development [92]. The exact function of IL-27 in RA progress and development is unknown due to its contradictory effects. However, some reports emphasize that this cytokine may induce pathways that can cause a milder disease, such as by promoting TH1 and suppressing TH2 and Th17 differentiation [97], blocking RANKL surface expression, and possibly restricting both neutrophil and γδ T cell accumulation [98]. Trials made on both murine [37] and cell [51] RA models have shown that curcumin decreases the production of IL-6.

### 4.6. IL-23 and IL-17 Axis

IL-23 is a cytokine with a pivotal role in disease perpetuation. In RA, this cytokine is mainly secreted by antigen-presenting cells and activated FLS [99,100], and probably by synovial macrophages [100]. Proinflammatory properties of IL-23 come mainly from its ability to vastly increase IL-17 secretion by stimulating survival, differentiation, and expression of Th17 cells [99,100,101] and, possibly, innate immune cells [102]. Presently, there is a lack of profound data on how curcumin could affect IL-23 expression in RA models; however, it has been shown that in a mouse model of psoriasis, this phytochemical can alleviate the production of that cytokine [103].

IL-17 is a pleiotropic cytokine suspected of a crucial role in establishing autoimmune diseases such as psoriasis or rheumatoid arthritis. IL-17 cytokine group consists of six subtypes ranging from A to F [104] with IL-17A being the most “infamous” due to its proinflammatory properties; it is usually seen as a group representative. IL-17A and its close homolog IL-17F are produced mainly by IL-23-stimulated Th17 cells, neutrophils and some populations of innate-like lymphocytes and acts synergistically with many other mediators activating a wide range of cell responses [101]. In rheumatoid arthritis, IL-17 is considered as one of the central cytokines involved in disease development. According to recent studies, the most of RA-promoting properties of IL-17 occurs from its synergistic effects with other cytokines (most importantly with TNF-α [105]) during the early stages of affliction establishment, forming self-perpetuating loops of pro-inflammatory activators and effectors [106]. IL-17 increases the production of IL-1β, IL-6, IL-8, IL-23, TNF-α, PGE2 and GM-CSF [99,100,101,105,107]. IL-17 also enhances those harmful interactions via recruitment and maintenance of inflammatory cells such as neutrophils, T cells or dendritic cells [101]. Moreover, IL-17 promotes bone and cartilage destruction by stimulating osteoclastogenesis (directly or via RANKL dependent pathway) [100] and release of MMP-1-2,-9 and -13 by synoviocytes and chondrocytes [107]. Furthermore, IL-17 promotes synovial neoangiogenesis (via enhancing VEGF production) and pannus expansion, probably even by inhibiting FLS apoptosis due to increasing their autophagy capabilities and boosting up anti-apoptotic [108] and reducing pro-apoptotic genes expression [107]. On the other hand, those discrepancies could be caused by the possibility that IL-17 is required majorly during the early stages of the disease, being nearly redundant in later phases [106]. Its whole dire properties came from synergistic interactions, because IL-17 alone is insufficient to cause a profound impact [104]. Nevertheless, IL-17 function in RA is definitely worth further investigation. Some studies have demonstrated that curcumin can inhibit IL-17 generation, not only in different autoimmune diseases like psoriasis [109] but also in collagen-induced arthritis (CIA) rat model [34].

### 4.7. Selected Cytokines of IL-2R Group

The next group of mediators involved in RA development consists of some cytokines from IL-2R group sharing a common γ chain, including IL-7, IL-15 and IL-21 [110].

IL-7 is known for being involved in osteoclastogenesis, probably mainly by its effect laid on naïve myeloid cells. This cytokine is stimulating their remodelling into pro-inflammatory M1 type macrophages and further forces osteoclast formation probably through increasing T, B cells and FLS RANKL expression and enhancing TNF- α and IL-6 induction [111]. Additionally, IL-7 promotes recruitment of monocytes/macrophages to endothelium [112] and boosts up their production of TNF-α, IL-1α, IL-1β, IL-6, CCL2, CCL5, MIP-1β and nitric oxide synthases (NOS) [111,113] exacerbating inflammation. Moreover, IL-7 may also be involved in inducing proliferation, differentiation and activation of TH1 and Th17 cells [78,111] and enhancing expression of their related cytokines [112,113]. Interestingly, some research reports that IL-7 activity is utterly TNF- α (and to a lesser extent IFN-γ) dependent due to IL-7 receptor expression induced by these cytokines [111].

Hitherto, the exact role of IL-15 in RA pathogenesis does not seem to be crystal clear. Nonetheless, its levels are elevated during disease [114] and strongly correlate with rheumatoid factor and ACPA levels [115,116]. Additionally, this interleukin is suspected of taking part in osteoclastogenesis [117], T cells activation and trafficking [115,116] and stimulating cytokines production. Studies have shown that blockade of IL-15 signal transduction leads to the limitation of autoreactive T cells response and proliferation and decreases in IL-1β, IL-6, IL-17, MMP-3, and, most importantly TNF-α [116]. Indeed, there are premises that IL-15 may precede TNF-α in the cytokine cascade and may even significantly stimulate expression of this cytokine [116] with all further consequences, presenting interleukin 15 as a promising therapeutic target.

IL-21 produced mainly by Th17 and Tfh cells favors activation, migration, and proliferation of FLS and immune cells [114]. This cytokine is known for regulating the functioning of Th17, Tfh, and B cells, macrophages, plasma cells from germinal centers, and plasmablasts [110,114,118,119]. As a result, increasing autoantibody and proinflammatory cytokine [110,114] production and exacerbating autoimmune inflammation are observed. Furthermore, IL-21 may participate in bone and joint destruction by promoting osteoclastogenesis and production of MP-1, -2, -3, -9, -13, and cathepsin K [110,118]. According to some research, the blockade of IL-21 signaling decreases synovitis, the cartilage damage score and can probably reduce infiltration of inflammatory cells even in established RA models, which could be a promising strategy for RA treatment [110]. A wide variety of published studies have indicated that curcumin inhibits the production of IL-7, IL-17, and IL-21 [52,120,121,122].

### 4.8. IL-8

IL-8 (also known as CXCL8) is a potent chemoattractant belonging to the CXC chemokine family [123,124]. In RA, IL-8 is probably responsible for recruiting cells into sites of inflammation [125], neutrophil activation, promoting their degranulation, and also release of superoxide and lysosomal enzymes, elevating cartilage damage and bringing pain [126]. Correspondingly, IL-8 may be involved in osteoclastogenesis [124] and angiogenesis [123]. Many studies have reported that curcumin inhibits the production of IL-8 in various in vitro and in vivo models of inflammation [127,128].

### 4.9. GM-CSF

Granulocyte-macrophage colony-stimulating factor (GM-CSF) is a cytokine that is extensively involved in RA development. GM-CSF is produced by many groups of cells, including some types of lymphocytes, vascular endothelial cells, and tissue-resident cells such as those in the synovium [76,129]. The main function of GM-CSF is to oversee the general functioning of myeloid-derived cells, monocytes, macrophages, and neutrophils by regulating their proliferation, maturation, survival, chemotaxis, adhesion, phagocytosis, and proinflammatory cytokine expression profile [129]. Potential GM-CSF contribution to RA involves augmentation of macrophage polarization to the M1 type, induction of proinflammatory response in CD4+ T, Th17, stromal cells, and ILCs, boosting up IL-1β, IL-6, IL-17, IL-23 production [129,130]. GM-CSF can also increase neutrophil gelatinase-associated lipocalin production, activating further immunologic responses and reducing chondrocyte proliferation [130]. Moreover, the presence of this cytokine can turn monocyte-derived dendritic cells (MoDCs) into a more aggressive IL-10-resistant phenotype and lead to their recruitment into the synovial fluid and tissue. MoDCs can further produce various proinflammatory cytokines, including TNF-α, IL-6, IL-12, and stimulate T and B cells [130]. Furthermore, this multifunctional mediator participates in rheumatoid arthritis exacerbation and causes nociceptor activation, bringing pain [76]. Regarding the previous studies, the treatment focused on blocking GM-CSF signaling significantly reduced RA, especially in combination with IL-17 blockade, and brought promising results as a potential alternative therapy, particularly for anti-TNF-α-resistant patients [129]. No studies have been conducted on the effect of curcumin on the granulocyte macrophage-colony stimulating factor in RA; however, it has been shown that a low dose of curcumin downregulates the GM-CSF mRNA level in human peripheral blood mononuclear cells, which are associated with RA [131,132].

### 4.10. IFN-γ

IFN-γ is another cytokine that seems to play an ambiguous role in RA, potentially acting in pro- and anti-inflammatory ways [133]. The exact reasons for those discrepancies remain mostly unknown; however, according to some papers, this mediator’s activity may depend on the patients’ individual predisposition or stage and severity of the disease [134]. IFN-γ is known to partially exhibit both bone-damaging and bone-protecting properties. Due to the ability to directly inhibit RANKL–RANK signaling via reducing RANKL levels, IFN-γ can block osteoclastogenesis; nonetheless, an abundance of RANKL can negate this effect. Alternatively, IFN-γ can also boost up levels of osteoclastic factors (including RANKL and TNF-α) and chemokines (CXCL10) by activating their production in immune cells [133,134] and takes part in macrophage differentiation leading to bone resorption. IFN-γ (mainly derived from B cells) probably inhibits Treg differentiation, suppressing immunoregulation and further exacerbating disease progression. However, this phenomenon presumably also depends on the stage of disease, occurring mostly during later phases [135]. Furthermore, proinflammatory effects generated by IFN-γ take part in MHCII induction activating immune cells, stimulate cytokines and reactive oxygen species, and regulate cell growth and survival [134]. Despite the fact that no studies have evaluated the effect of curcumin on IFN-γ in RA in vitro or in vivo, recent evidence has shown that IFN-γ production by T cells of psoriatic arthritis patients is significantly decreased [109].

### 4.11. TNF-α

TNF-α is a well-known proinflammatory molecule transducing a wide range of signal cascades as a master cytokine in various diseases, including rheumatoid arthritis. This potent mediator is produced by a vast amount of cells relevant for RA: monocytes, macrophages, fibroblasts, dendritic cells, and B and T cells [136]. In RA, TNF-α promotes different molecules’ expression: IL-1, IL-6, IL-8, MMPs, prostanoids, GM-CSF [124,125], etc., participating in the regulation of angiogenesis, pannus formation, cell adhesion, migration, and overall cell functioning under inflammatory conditions [136]. TNF-α also takes part in bone destruction by promoting osteoclastogenesis and suppressing bone formation by increasing Wnt signaling inhibitors (DKK1 and sclerostin) [137]. Despite being already present in some RA therapies and achieving good therapeutic results, blockade of TNF-α signaling cannot be universally and commonly used due to a large group of patients not responding to this type of treatment [138,139] because of differences in disease phenotypes. For this very reason, further investigations in finding new potential RA therapeutics are in dire need. A study published by Dai et al. in 2018 showed that curcumin inhibited the mTOR pathway and, subsequently, the production of TNF-α both in the serum and the synovium of RA rats [54].

## 5. Conclusions

Despite scientific support of potential benefits, curcumin is not used as a standard treatment option for rheumatoid arthritis. However, many studies have indicated that curcumin can modulate the immune response of the cells participating in the course of RA. This review highlighted and collected scientific evidence of the potential effect of curcumin on immune cells, on the cytokines secreted by them, as well as on several in vivo RA models. Notwithstanding the significant amount of studies reported in this review, the mechanisms of action of curcumin and its effect on cell populations that play a major role in RA require deeper investigation. Moreover, additional clinical studies are necessary to draw any definite conclusions on the efficacy of curcumin in the treatment of RA. However, despite the lack of significant data from clinical trials, in vitro and in vivo experiments have shown that curcumin can suppress the expression of inflammatory mediators and modulate immune cells, alleviating the course of RA. For this reason, curcumin’s immunomodulatory and anti-inflammatory role seems to have the potential to improve RA patients’ life naturally.

## Data Availability

The authors confirm that the data supporting the findings of this study are available within the article.
